# Alteration of Mesopontine Cholinergic Function by the Lack of KCNQ4 Subunit

**DOI:** 10.3389/fncel.2021.707789

**Published:** 2021-07-26

**Authors:** T. Bayasgalan, S. Stupniki, A. Kovács, A. Csemer, P. Szentesi, K. Pocsai, L. Dionisio, G. Spitzmaul, B. Pál

**Affiliations:** ^1^Department of Physiology, Faculty of Medicine, University of Debrecen, Debrecen, Hungary; ^2^Instituto de Investigaciones Bioquímicas de Bahía Blanca (INIBIBB), Consejo Nacional de Investigaciones Científicas y Técnicas (CONICET), Universidad Nacional Del Sur (UNS), Bahía Blanca, Argentina; ^3^Departamento de Biología, Bioquímica y Farmacia, Universidad Nacional del Sur, Bahía Blanca, Argentina

**Keywords:** KCNQ4 (Kv7.4), M-current, pedunculopontine nucleus, neuronal synchronization, non-syndromic hearing loss (DFNA2), potassium channels

## Abstract

The pedunculopontine nucleus (PPN), a structure known as a cholinergic member of the reticular activating system (RAS), is source and target of cholinergic neuromodulation and contributes to the regulation of the sleep–wakefulness cycle. The M-current is a voltage-gated potassium current modulated mainly by cholinergic signaling. KCNQ subunits ensemble into ion channels responsible for the M-current. In the central nervous system, KCNQ4 expression is restricted to certain brainstem structures such as the RAS nuclei. Here, we investigated the presence and functional significance of KCNQ4 in the PPN by behavioral studies and the gene and protein expressions and slice electrophysiology using a mouse model lacking KCNQ4 expression. We found that this mouse has alterations in the adaptation to changes in light–darkness cycles, representing the potential role of KCNQ4 in the regulation of the sleep–wakefulness cycle. As cholinergic neurons from the PPN participate in the regulation of this cycle, we investigated whether the cholinergic PPN might also possess functional KCNQ4 subunits. Although the M-current is an electrophysiological hallmark of cholinergic neurons, only a subpopulation of them had KCNQ4-dependent M-current. Interestingly, the absence of the KCNQ4 subunit altered the expression patterns of the other KCNQ subunits in the PPN. We also determined that, in wild-type animals, the cholinergic inputs of the PPN modulated the M-current, and these in turn can modulate the level of synchronization between neighboring PPN neurons. Taken together, the KCNQ4 subunit is present in a subpopulation of PPN cholinergic neurons, and it may contribute to the regulation of the sleep–wakefulness cycle.

## Introduction

The neuronal M-current is a voltage-gated non-inactivating potassium current that sets resting membrane potential, regulates excitability, and shapes action potential firing (Delmas and Brown, 2005; Brown and Passmore, 2009). In the presynaptic location, it controls synaptic vesicle release (Huang and Trussell, 2011). It is under the regulation of several neuromodulatory actions; probably the best-known pathway is the cholinergic inhibition through muscarinic acetylcholine receptors (Brown and Adams, 1980; Marrion, 1997; Hernandez et al., 2008). The KCNQ2 to KCNQ5 (Kv7.2-5) channel subunits are responsible for the M-current. They belong to the superfamily of voltage-gated potassium channels and can form homo- or heterotetrameric channels (Brown and Passmore, 2009). In the central nervous system (CNS), many brain areas express KCNQ2, KCNQ3, and KCNQ5, whereas KCNQ4 is restricted to certain nuclei of the auditory brainstem, such as the cochlear nuclei, nuclei of the lateral lemniscus, and the inferior colliculus (Kharkovets et al., 2000; Delmas and Brown, 2005; Brown and Passmore, 2009). Other areas of the brainstem that express KCNQ4 are the principal and spinal trigeminal nuclei and members of the reticular activating system (RAS) such as the raphe nuclei and the ventral tegmental area (VTA) (Kharkovets et al., 2000; Koyama and Appel, 2006; Hansen et al., 2008).

Mutations in the *KCNQ4* gene lead to an autosomal-progressive non-syndromic hearing loss due to the degeneration of the outer and, in a lesser extent, inner hair cells of the cochlea, known as DFNA2 (De Leenheer et al., 2002; Nie, 2008). Transgenic mice that either expressed a human KCNQ4 mutation or lacked KCNQ4 channel expression serve as models of the disease (Kharkovets et al., 2006; Carignano et al., 2019). Additionally, this mutation alters somatosensory functions due to the lack of expression in skin somatosensory receptors and dorsal root ganglia (DRG) both in human and mouse (Heidenreich et al., 2011).

Interestingly, some KCNQ4-positive brainstem nuclei overlap with cholinoceptive areas (Kharkovets et al., 2000; Woolf and Butcher, 2011). Considering that cholinergic signaling modulates the M-current, it is possible that this pathway regulates neuronal excitability of the target areas (Delmas and Brown, 2005; Brown and Passmore, 2009). In this regard, it was recently shown that the KCNQ4-mediated M-current contributes to neuromodulatory autoregulation (Su et al., 2019). Therefore, we sought evidence for the hypothesis that the M-current formed by the KCNQ4 subunit is present on another important member of the RAS, the mesopontine cholinergic neurons regulating its activity.

One of the main mesopontine cholinergic areas is the pedunculopontine nucleus (PPN), which is not only the source of cholinergic fibers but also receives cholinergic inputs from the neighboring laterodorsal tegmental nucleus (LDT), the contralateral PPN, and local cholinergic axon collaterals (Honda and Semba, 1995; Mena-Segovia et al., 2008). The PPN has cholinergic and non-cholinergic (GABAergic and glutamatergic) neurons, which show different activity patterns during global brain states, such as slow-wave sleep (SWS), paradoxical sleep (PS), and wakefulness (W). Altogether, PPN units are synchronized during SWS, but the level of synchronization is reduced during PS and W (Petzold et al., 2015; Mena-Segovia and Bolam, 2017). Since the neuronal network is not fully understood, the mechanism of this synchronization remains unclear.

We have previously shown that almost all PPN cholinergic neurons possess the M-current, whereas GABAergic neurons lack it. The M-current of the cholinergic neurons is responsible for the after hyperpolarization current and spike frequency adaptation (SFA) and is capable of modulating high threshold membrane potential oscillations (Bordas et al., 2015). In addition, cholinergic neurons facilitate transitions to wake and rapid eye movement (REM) sleep, which would work in concordance with glutamatergic neurons to induce wakefulness (Kroeger et al., 2017; Mena-Segovia and Bolam, 2017). However, there are still some pending questions regarding the M-current of the PPN, such as the contribution of each individual subunit to the molecular composition of the KCNQ channels and its functional implications. The most abundant subunits in the CNS are KCNQ2 and KCNQ3, located mainly in the axon initial segments and nodes of Ranvier (Devaux et al., 2004). In addition, the KCNQ5 subunit is highly expressed; however, its cellular and/or subcellular localization differs from that of the KCNQ2 and KCNQ3 subunits (Huang and Trussell, 2011). KCNQ4 is the least abundant subunit in the CNS. It is present in the auditory brainstem nuclei and some members of the RAS. Its contribution to and its function in these two systems are still unknown. On the other hand, in the auditory brainstem, KCNQ4 may participate in sound processing and cochlear modulation; in the RAS, it could modulate changes in the brain states associated with SWS, PS, and W.

We aimed to demonstrate the physiological significance of the M-current in the PPN and the contribution of KCNQ4 to its activity. We have shown that KCNQ4 knockout (KO) mice displayed alterations in the activity cycles and demonstrated the presence of KCNQ4 in a subgroup of cholinergic neurons and the changes in the expression of the KCNQ3 subunit by deletion of KCNQ4. We also found that activation of the cholinergic inputs of the PPN can inhibit its M-current and that the M-current contributes to the synchronization of neighboring neurons. Our findings add new roles for the CNS-expressed KCNQ4 channel, such as modulation of the PPN activity, which may affect the activity cycles. As the expression of this subunit is restricted to certain brainstem nuclei, subunit-specific modulators can potentially act as medication for disturbances in the sleep–wakefulness cycle.

## Materials and Methods

### Solutions and Chemicals

For electrophysiological experiments, artificial cerebrospinal fluid (aCSF) was used in the composition below (in millimolars): NaCl, 120; KCl, 2.5; NaHCO_3_, 26; glucose, 10; myo-inositol, 3; NaH_2_PO_4_, 1.25; sodium pyruvate, 2; CaCl_2_, 2; MgCl_2_, 1; ascorbic acid, 0.5; pH 7.4. For the preparation of slices, low-Na^+^ aCSF was administered. In this solution, 95 mM NaCl was replaced by sucrose (130 mM) and glycerol (60 mM). All chemicals were purchased from Sigma (St. Louis, MO, United States), unless stated otherwise.

### Mouse Models

Animal experiments were conducted in accordance with the appropriate national and international laws (EU Directive 2010/63/EU for animal experiments) and institutional guidelines on the care of research animals. The experimental protocols used below were approved by the Committee of Animal Research of the University of Debrecen (6/2011/DEMÁB, 5/2015/DEMÁB, and 19/2019/DEMÁB) and Universidad Nacional del Sur (083/2016). Ten- to 19-days-old mice were employed for slice electrophysiology, whereas 52–to 69-days-old mice were used for behavioral tests. Mice expressing the tdTomato fluorescent protein or ChR2 in a choline acetyltransferase (ChAT)-dependent manner (*n* = 59 and 5, respectively) and mice of both sexes expressing tdTomato in a type 2 vesicular glutamate transporter- (Vglut2; *n* = 13) or GAD65-dependent (type 2 glutamate decarboxylase; *n* = 11) manner were employed. In order to obtain mice for slice electrophysiology, homozygous floxed-stop-tdTomato [B6;129S6-Gt(ROSA)26Sor^tm9(CAG–tdTomato)Hze/^J; Jax mice accession no. 007905], ChAT-cre [B6;129S6-Chat^tm2(cre)Lowl/^J; Jax no. 006410], and Vglut2-cre [*Slc17a6*^*tm2(cre)Lowl*^, also called Vglut2-ires-Cre; Jax no. 028863] or homozygous floxed-stop-channelrhodopsin-2 [B6;129S-Gt(ROSA)26Sortm32.1(CAG-COP4^∗^H134R/EYFP)Hze/J] and ChAT-cre (see above) strains purchased from Jackson Laboratories (Bar Harbor, ME, United States) were crossed in our animal facility. The KCNQ4 KO strain (*Kcnq4*^–/–^) was provided by Prof. Thomas Jentsch (Kharkovets et al., 2006). Heterozygous animals were bred in the animal facility of the Department of Physiology, University of Debrecen, and that of the INIBIBB, Universidad Nacional del Sur. Young pups were genotyped, and KO and wild-type (WT) animals were included in the experiments.

### Electrophysiology

Coronal midbrain slices (200 μm thick) were prepared in ice-cold (approx. 0 to –2°C) low-Na^+^ aCSF with a Microm HM 650 V vibratome (Microm International GmbH, Walldorf, Germany). The slices were incubated in normal aCSF at 37°C for 1 h before starting the recording. Patch pipettes with resistance of 6–8 MΩ were fabricated and filled with internal solution with the following composition (in millimolars): K-gluconate, 120; NaCl, 5; 4-(2-hydroxyethyl)-1-piperazineethanesulfonic acid (HEPES), 10; Na_2_-phosphocreatinine, 10; EGTA, 2; CaCl_2_, 0.1; Mg-ATP, 5; Na_3_-GTP, 0.3; biocytin, 8; pH 7.3. Whole-cell patch-clamp experiments were conducted at room temperature (22–25°C) on neuronal somata with an Axopatch 200 A amplifier (Molecular Devices, Union City, CA, United States). Clampex 10.0 software (Molecular Devices, Union City, CA, United States) was used for data acquisition and Clampfit 10.0 (Molecular Devices) software for data analysis. Only stable recordings with minimal leak currents were considered; recordings with series resistance below 20 MΩ for the voltage-clamp and 30 MΩ for the current-clamp experiments with less than 10% change were included.

Both voltage- and current-clamp configurations were used. In certain experiments, 1 μM tetrodotoxin (TTX; Alomone Laboratories, Jerusalem, Israel) was used to eliminate action potential generation. For blockade of the M-current, 20 μM XE991 [10,10-*bis*(4-pyridinylmethyl)-9(10*H*)-anthracenone dihydrochloride] (Tocris Cookson Ltd., Bristol, United Kingdom) was used. M-current openers, such as the nonspecific retigabine, the KCNQ2- and KCNQ4-specific ML213 {*N*-(2,4,6-trimethylphenyl)-bicyclo[2.2.1]heptane-2-carboxamide}, and the KCNQ2- and KCNQ3-specific ICA27243 [*N*-(6-chloro-pyridin-3-yl)-3,4-difluoro-benzamide] (20 μM; Tocris Cookson Ltd., Bristol, United Kingdom) were administered in certain experiments (Wickenden et al., 2008; Gunthorpe et al., 2012; Linley et al., 2012; Brueggemann et al., 2014).

The protocols detailed below were used to assess the presence of the M-current or the functional consequences of the M-current on PPN cholinergic (and, in some cases, glutamatergic) neurons. For determining spike train properties, the current-clamp configuration was used. For recording of the M-current, the voltage-clamp configuration was used. Neurons were held on –20 mV holding potential, and 1-s-long repolarizing steps were employed from –30 to –60 mV with a 10-mV decrement. Recordings were performed with TTX, except when the LDT was stimulated with optogenetic methods. For the detection of the spike train pattern, 1-s-long square current pulses were used between –30 and +120 pA with a 10-pA increment in the current-clamp configuration. The resting membrane potential was set to –60 mV. An adaptation index (AI) was calculated using the following formula: AI = 1 – (*F*_last_/*F*_initial_), where *F*_last_ is the frequency of the last two action potentials and *F*_initial_ is the average frequency of the first three action potentials. Sweeps with 100 pA depolarizing square current injection were included in the analysis. Only those recordings where at least eight action potentials were seen in the control were considered. In those cases, where this number decreased below five due to the robust action of the M-current openers, the AI was considered as 1.

For synchronization analysis, the neighboring but synaptically non-coupled cholinergic neurons were patched and both neurons were simultaneously depolarized by using 1-s-long depolarizing square current injections with an amplitude of 100 pA in the current-clamp configuration. Absolute values of time differences between action potentials were plotted using 20-ms bins. Individual graphs under the control conditions and after the application of XE991 were fitted with a single exponential function: *y* = *a* + *b*^∗^*e*^(τ^∗^*x*)^). The parameter *τ* was used as a measure of synchronicity.

In experiments with optogenetics, 500-μm-thick coronal midbrain slices were cut including the LDT and the PPN. An optical fiber was administered to the LDT and 500-ms-long illuminations with 470 nm wavelength and 1 Hz frequency were used. Before and in parallel with it, whole-cell patch-clamp recordings of the M-current (see above) were performed on PPN cholinergic neurons.

Visualization of the tdTomato and enhanced yellow fluorescent protein (EYFP) fluorescent markers was performed using a wide-field fluorescent imaging system (Till Photonics GmbH, Gräfeling, Germany) containing a xenon bulb-based Polychrome V light source, a CCD camera (SensiCam, PCO AG, Kelheim, Germany), an imaging control unit, and the Till Vision software (version 4.0.1.3).

### Morphological Identification of the Investigated Neurons

Neurons were labeled with biocytin during the patch-clamp recording and slices were fixed (4% paraformaldehyde in 0.1 M phosphate buffer, pH 7.4, 4°C) for morphological analysis of the neurons. *Tris*-buffered saline (8 mM *Tris* base, 42 mM *Tris*ma–HCl, 15 mM NaCl, pH 7.4) supplemented with 0.1% Triton X-100 and 10% bovine serum (60 min) was applied for permeabilization. For recovery, the samples were incubated in streptavidin-conjugated Alexa 488 (1:300; Molecular Probes Inc., Eugene, OR, United States) dissolved in phosphate buffer for 90 min.

After the recovery procedure, the neurons were visualized with a confocal microscope (Zeiss LSM 510; Carl Zeiss AG, Oberkochen, Germany). Tile scan images were taken with ×40 objective and with 1-μm optical slices.

### Immunohistochemistry

The immunofluorescence of KCNQ2 to KCNQ5 and ChAT was performed on 15-μm coronal brain sections from the cryostat of adult (3–6 months old) transcardially perfused WT, KCNQ4 KO, or ChAT-tdTomato mice. Brain slices were post-fixed in 4% paraformaldehyde and then were permeabilized with 2% Nonidet and 1% bovine serum albumin (BSA) for 1 h. For the detection of KCNQ2, KCNQ3, and KCNQ5, a retrieval protocol was necessary before blocking. The slices were treated with 0.3 M glycine in phosphate-buffered saline (PBS) for 30 min at room temperature and then incubated with 10 mM citrate buffer (pH 6) for 30 min at 80°C. Primary antibody goat anti-ChAT (1:100; #AB144P, EMD Millipore, Burlington, MA, United States) was incubated for 48 h with either rabbit anti-KCNQ2 (1:200; #APC-050, Alomone Labs, Jerusalem, Israel), rabbit anti-KCNQ3 (1:100; #APC-051, Alomone Labs, Jerusalem, Israel), rabbit anti-KCNQ4 (1:400), or guinea pig anti-KCNQ5 (1:200) (Spitzmaul et al., 2013). Donkey anti-goat Alexa Fluor 546, donkey anti-guinea pig Alexa Fluor 488, and donkey anti-rabbit Alexa Fluor 488 antibodies (Vector Laboratories Inc., Burlingame, CA, United States) were used for another 24 h of incubation. The nuclei were visualized with DAPI. For each KCNQ subunit antibody, a control protocol without primary antibody was performed. Fluorescent images were taken with Zeiss LSM 900 confocal microscope (Carl Zeiss AG, Jena, Germany). For immunofluorescence (IF), we used four WT, four ChAT-tdTomato, and three KCNQ4 KO mice.

### RNA Extraction, Reverse Transcription, and qPCR

Coronal midbrain blocks were prepared and areas containing the PPN were taken out from adult (15–30 weeks old) mice. For each experiment, samples from three to four mice were pooled. Total RNA was extracted from PPN using the TransZol reagent (TransGen Biotech, Beijing, China) in combination with the Direct-Zol RNA mini prep kit (Zymo Research, Irvine, CA, United States). Complementary DNA (cDNA) was produced from 500 ng of total RNA with EasyScript Reverse Transcriptase (cat. #AE101, TransGen Biotech) using anchored oligo(dT)s following the manufacturer’s indications. Quantitative PCR (qPCR) was carried out using the cDNA generated previously employing the SensiFAST SYBR mix No-ROX Kit (Bioline, London, United Kingdom) in a Rotor-Gene 6000 real-time PCR cycler (Qiagen, Germany). As reference genes, we used *GAPDH* and *HPRT*. Messenger RNA (mRNA) subunit expression was referred to the geometric mean of *GAPDH* and *HPRT*. Table 1 shows the list of primers used for PCR. Data analysis was done applying the ΔΔC^*t*^ method (Livak and Schmittgen, 2001; Schmittgen and Livak, 2008) to obtain the relative mRNA quantification (RQ).

**TABLE 1:** Primers used for qPCR experiments.

Primer name	Forward	Reverse
KCNQ2	GGGGCCCAACAATAACGGAT	TTTCTCCACCTTCCCAAGCC
KCNQ3	CGCGCTTGTGTTCCTGATTG	CAGCCCAGATCCTCAAAGCA
KCNQ4	TATGGTGACAAGACGCCACAT	GCTTCTCAAAGTGCTTCTGCC
KCNQ5	ATTGGCTATGGAGACAAAACACC	CGGTGCTGCTCCTGTACTTTT
HPRT	GTTCTTTGCTGACCTGCTGGA	ACCCCCGTTGACTGATCATT
GAPDH	GAGAAACCTGCCAAGTATGATGAC	ATCGAAGGTGGAAGAGTGGG
ChAT	AAGTCCCTGCAGTTTGTGGT	TTCTGGGAGCAGGGAGTTCA

### Activity Wheel Test

To evaluate the circadian locomotor activity rhythms, we housed mice individually in cages equipped with an activity wheel (Campden Instruments Ltd., Loughborough, United Kingdom). Young adult (52–69 days old) KCNQ4 KO mice (*n* = 16) and WT littermates (*n* = 16) were used because KCNQ4 KO mice develop a fast hearing loss and the activity wheel performance changes with age (Kharkovets et al., 2006; Fodor et al., 2020). The background strain for the KCNQ4 KO mice is a mixed between the C57BL/6J and C3H/HeJ strains. The last one carries the *rd1* allele, which, in a homozygous condition, develops retinal degeneration (The Jackson Laboratory stock no. 000659). We tested for the presence of the *rd1* allele by genotyping randomly in five animals that exhibited only the *wt* allele (data not shown). Additionally, to check for the integrity of the visual pathway, we altered the circadian rhythm by changing the light/dark cycles to generate a bifurcation in their rhythms in order to accommodate two-light and two-night periods (LDLD) of 6 h each (Gorman and Elliott, 2003; Harrison et al., 2016). The animals had voluntary and unlimited access to the activity wheel and were placed in a room for 7 days to accommodate the alternating 6-h illumination and 6-h darkness periods. After accommodation, we recorded their activity for 5 days with the same conditions of illumination (LDLD conditions). After that, we recorded the activity in the free-running condition (DD) during 5 days of complete darkness. We analyzed the last 3 days of recordings and determined the duration of activity cycles and the distances moved per day. Period time was determined by the Lomb–Scargle periodogram^1^. Due to technical problems, three of the KO and one of the WT animals were taken out of the study; thus, 13 KO and 15 WT mice were included.

### Statistics

All data represent the mean ± SD. The normal distribution of the datasets was evaluated with the D’Agostino and Pearson omnibus normality test. Paired Student’s *t*-test, one-way ANOVA, and *post-hoc* Tukey’s multiple comparison test were applied to assess statistical significance for pairwise comparisons in the case of datasets with normal distribution, whereas Bonferroni’s multiple comparisons test was employed for multiple comparisons. Paired Student’s *t*-test was used in those experiments where the statistical test was not specified. Statistical analysis was performed using Microsoft Excel or SigmaPlot 12.0 and GraphPad Prism 5.01 (GraphPad software, San Diego, CA, United States). The significance levels were: ^∗^*p* < 0.050; ^∗∗^*p* < 0.010; ^∗∗∗^*p* < 0.001.

## Results

### Functional Role of KCNQ4 in the RAS

**FIGURE 1:**
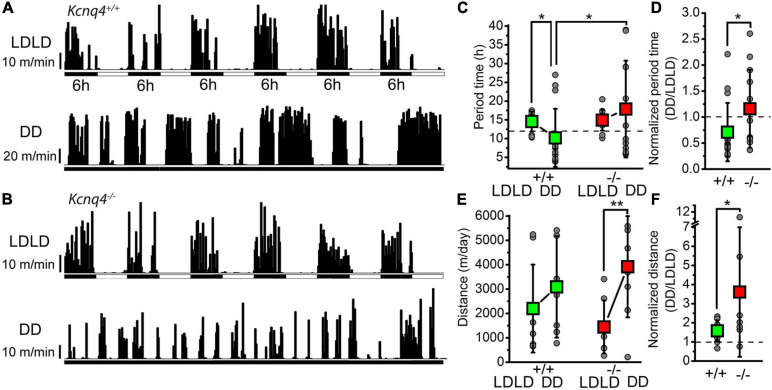
KCNQ4 knockout (KO) mice show alterations of the activity cycle adaptation to changes in light–darkness conditions with the activity wheel test. **(A)** Activity cycles recorded with a wild-type (WT) mouse (*Kcnq4*^+/+^) with 6-h alterations of light–darkness cycles (LDLD) and in complete darkness (DD). *Black vertical bars* represent the distances ran in 10-min bins. **(B)** Activity cycles with the same arrangement as in panel **(A)** for KCNQ4 KO mouse (*Kcnq4*^–/–^). **(C)** Statistical comparisons of the alterations in period length under the LDLD and DD conditions with WT (+/+) and KO (–/–) mice (*green squares*: WT; *red squares*: KO; *gray circles*: individual data). Under the LDLD conditions: 14.55 ± 2.64 h for WT and 14.92 ± 2.77 h for KO. Under the DD conditions: 10.2 ± 7.72 h for WT (*p* = 0.0242 compared with LDLD) and 17.88 ± 12.9 h for KO. The period times of WT and KO mice under the DD conditions were also statistically significant (*p* = 0.0331). *Dashed line* indicates 12 h. **(D)** Period times under the DD conditions normalized to the LDLD conditions of each case with the same arrangement as in panel **(C)** (*dashed line*: 1). The normalized value for the WT was 0.71 ± 0.56 and for the KO was 1.15 ± 0.76 (*p* = 0.0461). **(E)** Comparison of the distances ran in 3 days by WT (+/+) and KO (–/–) mice under the LD and DD conditions [the arrangement is the same as that in panel **(C)**] (significant difference at *p < 0.050; **p < 0.010). For the WT, the distance ran under LDLD was 2,203 ± 1,804 m/day and that under DD was 3,091 ± 2,083 m/day. For KO mice, the distance ran under LDLD was 1,445 ± 1,088 m/day and that under DD was 3,919 ± 2,075 m/day (*p* = 0.0049). **(F)** Distances ran under the DD conditions normalized to the LDLD conditions for each case with the same arrangement as in panel **(C)** (*dashed line*: 1). The normalized value for the WT was 1.58 ± 0.55 and that for KO was 3.6 ± 3.39 (*p* = 0.0482).

Firstly, we aimed to investigate whether KCNQ4 contributes to the regulation of the sleep–wakefulness cycle. To measure this, we evaluated the circadian locomotor activity by measuring voluntary wheel running in young adult KCNQ4 KO and WT mice (*n* = 13 and 15, respectively). In order to discard any potential impairment in the visual pathway, we induced mouse circadian bifurcation by altering the LD cycle to 6-h LDLD periods. The actogram exhibited two activity bouts during both scotophases for each genotype in this condition (Figures 1A,B), indicating the intactness of functions related to vision. The period time under the LDLD conditions showed no difference between genotypes (Figure 1C). Next, we studied the distance ran by both mouse genotypes in LDLD. Again, we did not find significant differences in the distance traveled between WT and KO mice in the LDLD conditions (Figure 1E).

To evaluate the intrinsic circadian rhythm in both genotypes, we performed an analysis of mice in the free-running or constant darkness conditions (DD). Compared to LDLD, in the DD conditions, the period time was significantly reduced in WT mice, but showed only a tendency to increase in KO mice (Figure 1C). Regarding the distance traveled in the DD conditions, for WT animals, we observed a tendency to increase, although it was not statistically significant compared to that LDLD. However, KO mice showed an approximately twofold increase in the distance traveled in the DD compared to the LDLD conditions (Figure 1E). In the DD conditions, all parameters showed a higher variability of individual measurements. Comparing both genotypes, the period time under the DD conditions was significantly longer in KO than in WT mice (Figure 1C); the distance traveled was not statistically significant (Figure 1E). When the period time under the DD conditions was normalized to the LDLD conditions in each case, this parameter was significantly greater in KO mice (Figure 1D). However, when the distances under the DD conditions were normalized on the LDLD conditions, this parameter was proven to be significantly greater in KO mice, with a much greater standard deviation (Figure 1F).

Taken together, the lack of KCNQ4 seems to have a mild but detectable impact on adaptation to changes in the LD cycle and on movement regulation related to activity cycles.

Several CNS nuclei control the sleep–wakefulness cycle. However, KCNQ4 expression is restricted to brainstem nuclei, including some members of the RAS such as the VTA and the raphe nuclei (Kharkovets et al., 2000; Koyama and Appel, 2006; Hansen et al., 2008). The PPN, as a RAS member, contributes to the regulation of the sleep–wake behavior. We determined the presence of the M-current mainly in cholinergic neurons (Bordas et al., 2015), and these neurons might regulate the transitions between brain states (Kroeger et al., 2017). In consequence, we further investigated the participation of KCNQ4 in the already known action of the M-current in neuronal PPN populations.

### Contribution of the KCNQ4 Channel Subunit to the M-Current in PPN Neurons

#### Neuronal Population Analysis

By using genetically labeled mice, we already demonstrated the presence of the M-current in cholinergic, but not GABAergic, neurons (Bordas et al., 2015). Presently, we have extended our analysis to glutamatergic neurons (Bordas et al., 2015). We analyzed the M-current in genetically labeled PPN glutamatergic, cholinergic, and GABAergic neurons. The M-current was present in almost all PPN cholinergic neurons but absent in the GABAergic ones (for cholinergic neurons: 30.5 ± 21.6 pA at –60 mV, 40.5 ± 25.4 pA at –50 mV, 45.4 ± 26.3 pA at –40 mV, and 39.4 ± 24.2 pA at –30 mV repolarizing steps; for glutamatergic neurons: 0.7 ± 1.4 pA at –60 mV, 2.36 ± 2.95 pA at –50 mV, 4.42 ± 6.1 pA at –40 mV, and 5.16 ± 6.5 pA at –30 mV repolarizing steps; for GABAergic neurons: 2.08 ± 2.8 pA at –60 mV, 4.03 ± 4.3 pA at –50 mV, 3.91 ± 2.4 pA at –40 mV, and 2.83 ± 2.88 pA at –30 mV repolarizing steps). The M-current amplitudes of cholinergic neurons were significantly different from those of GABAergic and glutamatergic ones (*p* < 0.0001), but no significant difference was found between the glutamatergic and GABAergic populations (*p* > 0.9999, one-way ANOVA with Bonferroni’s multiple comparisons test).

In glutamatergic neurons, 6.8% (*n* = 32) presented the M-current. One of these neurons was proven to be ChAT-positive, possibly belonging to the population of glutamatergic–cholinergic neurons (Gunthorpe et al., 2012). We concluded that the majority of non-cholinergic neurons lack the M-current, but very few exceptions may exist for the glutamatergic neuron population.

#### Expression of the KCNQ Subunits in the PPN

**FIGURE 2:**
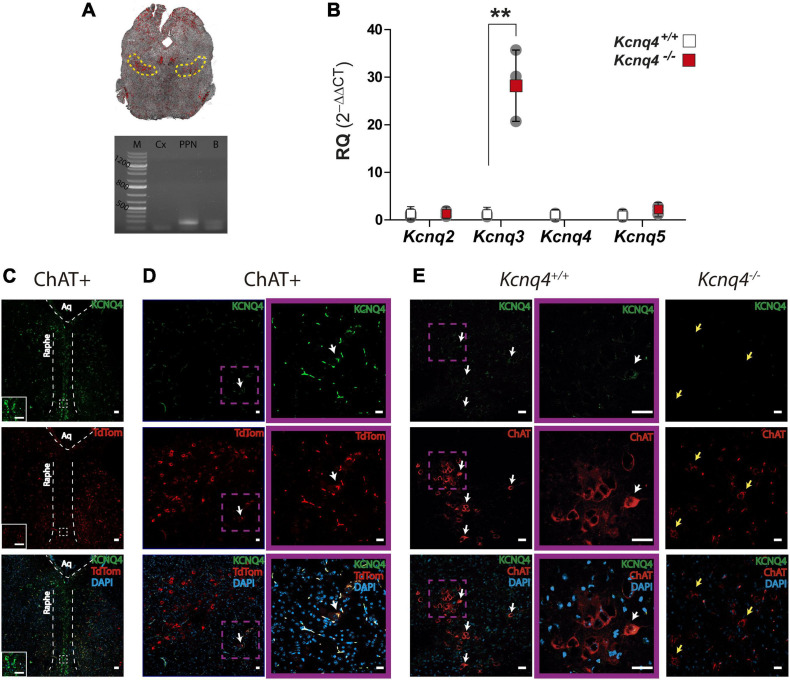
KCNQ subunits expression in the PPN.**(A)** *Top*: Confocal image of a coronal midbrain slice (–4.60 mm from bregma), showing the location of the PPN determined by ChAT labeling *(yellow dashed line)*, which corresponds to the area where tissue samples were collected. *Bottom*: Agarose gel showing PCR band corresponding to ChAT expression. *M*: molecular weight marker, *Cx*: brain cortex (negative control), *PPN*: sample containing the PPN, *B:* whole brain (positive control). **(B)** Relative quantification (RQ) of mRNA expression for each subunit from WT *(white)* and KO *(red)* mice. The fold change of each subunit was calculated using 2^–ΔΔCt^. Data represented as mean ± SD (*n* = 3). Student’s *t*-test; **p = 0.0028). **(C)** Brain coronal section of a ChAT-tdTomato mouse midbrain showing KCNQ4 staining in the raphe nucleus. The *inset* is a higher magnification of the section delimited by the *white square with dashed line*. *Scale bar*: 50 μm in both pictures. **(D,E)** KCNQ4 immunofluorescence on coronal brain sections of ChAT-tdTomato (ChAT+; **D**), WT (*Kcnq4*^+/+^; **E**), and KCNQ4 KO mice (*Kcnq4*^–/–^; **E**). Both models revealed that only a subpopulation of PPN cholinergic neurons located on the external limits possess KCNQ4 *(white arrows)*. Higher magnification of KCNQ4-positive neurons *(purple square)*. KCNQ4 could not be detected on PPN cholinergic neurons in KO animals *(yellow arrows)* that confirmed the specificity of the antibody. *Upper panel*: KCNQ4 *(green)*. *Middle panel*: ChAT immunolabeling or tdTomato expression under ChAT promoter *(red)*. *Bottom panel*: Merged image with DAPI. Scale bar: 20 μm.

We evaluated gene and protein expression of KCNQ channel subunits in midbrain tissues containing PPN for WT and KCNQ4 KO mice. For quantitative PCR (qPCR), we punched a block of midbrain containing mainly PPN (Figure 2A, top). To confirm the presence of the nucleus in the tissue samples, we search for mRNA of the cholinergic-neuron marker ChAT (Figure 2A, bottom). Samples exhibiting the proper band size for the ChAT complementary DNA (cDNA) were further analyzed. By qPCR analysis, we found the expression of the *Kcnq4* subunit in WT animals, together with *Kcnq2, -3*, and *-5*. In KCNQ4 KO animals, this expression profile changed, showing an increase of about 30-fold for *Kcnq3* subunit mRNA expression (*p* = 0.0028, Student’s *t*-test), while *Kcnq2* and *-5* showed no differences in their expression level between WT and KO animals (Figure 2B).

Next, we analyzed the presence and localization of KCNQ4 protein subunit as well as the neuronal subunits KCNQ2, -3, and -5 by immunofluorescence. We identified cholinergic neurons by using either anti-choline acetyltransferase (ChAT) antibody in WT mice or tdTomato expression in ChAT-Cre mouse strain. To control the specificity of the anti-KCNQ4 labeling, we stained raphe nucleus neurons from coronal section 68 (-4.48 mm from bregma) according to the Paxinos Atlas (Franklin and Paxinos, 2013), which were positive for KCNQ4 (Figure 2C). In both mouse strains, we found KCNQ4 expression in a subset of cholinergic neurons of the PPN (Figures 2D,E). In these neurons, KCNQ4 signal was present in the whole cytoplasm and in minor cases seems to be restricted to the somatodendritic surface membrane (Figure 2D). KCNQ4 is rather present in caudal than rostral locations, and not the whole cholinergic population exhibited KCNQ4-specific labeling, only laterally located subgroups (Figure 2E). KCNQ4 signal is absent in PPN section of KCNQ4 KO animals (Figure 2E, *n*=3), but the number of cholinergic neurons did not differ significantly from WT (143.3 ± 36.0/mm^2^ in WT and 125.5 ± 36.9/mm^2^ in KO; *p* = 0.2583; *n* = 4 for each genotype). After cell count, we determined that the proportion of KCNQ4-positive PPN cholinergic neurons was 9.0 ± 4.8% in WT and ChAT-tdTomato mice (*n* = 4 for each).

**FIGURE 3:**
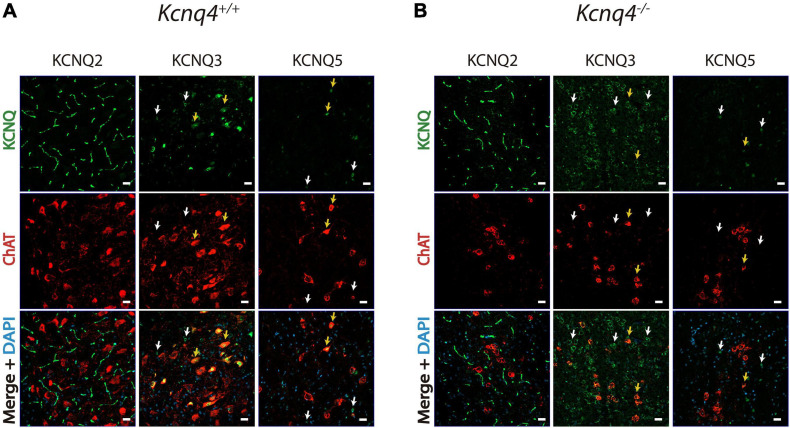
Expression of the other neuronal KCNQ subunits in the PPN. KCNQ2, KCNQ3, and KCNQ5 staining of coronal PPN sections in wild-type (WT, *Kcnq4^+/+^*) **(A)** and knockout (KO, *Kcnq4*^–/–^) **(B)** mice. KCNQ2 was found in neuron fibers without co-localization in cholinergic neurons. KCNQ3 and KCNQ5 were located in the soma of both cholinergic (yellow arrows) and non-cholinergic neurons. For KCNQ2 and KCNQ5, there was no difference between genotypes. Besides, KCNQ3 in KO animals labeled mostly non-cholinergic (white arrows) neurons. *Upper panel*: KCNQ subunit (*green*). *Middle panel*: ChAT (*red*). *Bottom panel*: merged with DAPI. *Scale bar*, 20 μm.

As KCNQ4 KO mice exhibited an altered gene expression profile of the neuronal KCNQ channel subunits in tissues containing PPN, we further investigated the expression pattern of KCNQ2, -3, and -5 protein by IF. In WT animals, we observed KCNQ2 expression in neuron fibers in the PPN region. However, its localization was different from that of cholinergic neurons (Figure 3A). On the contrary, KCNQ3 labeled most of the cholinergic neurons in the PPN. We obtained clear staining in the neuron soma. Besides, KCNQ3 labeled a few non-cholinergic neurons (Figure 3A, white arrows). Finally, we observed KCNQ5 staining in cholinergic neurons (Figure 3A, yellow arrows), but it is also present in certain non-cholinergic neurons (Figure 3A, white arrows). In KCNQ4 KO animals, KCNQ2 and 5 staining in the PPN showed difference neither in localization nor in signal intensity with the WT (Figure 3B). However, we observed a change in the expression pattern for KCNQ3 in KO animals. While it is still present in some cholinergic neurons from the PPN (Figure 3B, yellow arrows), the number of labeled-non-cholinergic neurons increase drastically (Figure 3B, white arrows). Negative control experiments in the absence of the primary antibody exhibited no fluorescence signal corresponding to either KCNQ2, KCNQ3, or KCNQ5 antibody in PPN sections (not shown).

In consequence, our studies showed a differential expression profile for KCNQ2 to -5 subunits in the PPN and an alteration of this pattern in PPN neurons by deletion of KCNQ4 subunit mostly altering KCNQ3 subunit expression.

#### M-Current Properties in KCNQ4 KO Mice

**FIGURE 4:**
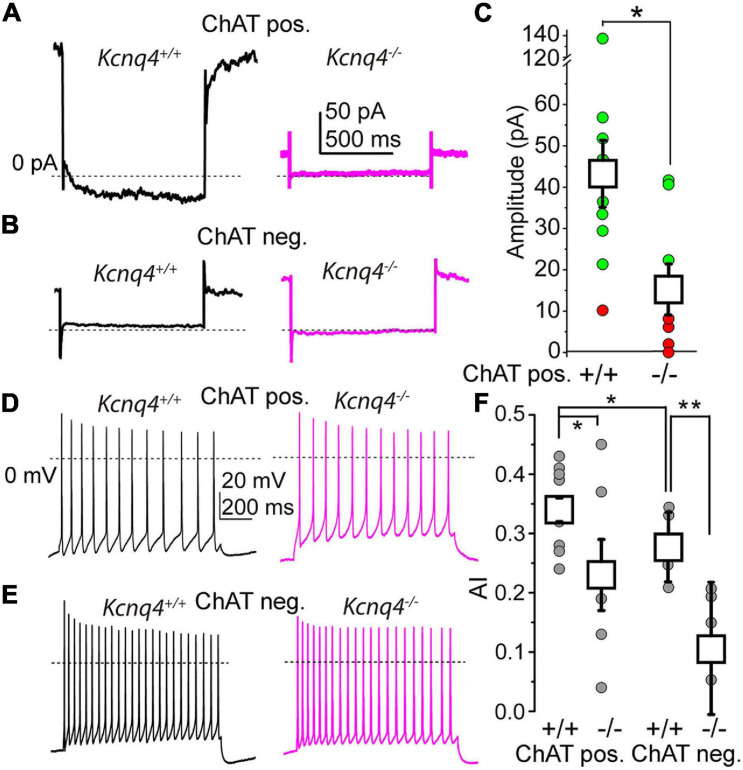
More than 60% of the cholinergic neurons lack the M-current in KCNQ4 knockout (KO) samples. **(A)** Representative current traces from cholinergic neurons of a wild-type (WT; *Kcnq4^+/+^*, *black*) and a knockout (KO; *Kcnq4*^–/–^, *purple*) sample elicited by a 1-s-long repolarizing step from –20 mV holding potential to –40 mV. The mean values were 175.97 ± 27.67 pA for the WT and 66.95 ± 19.8 pA for the KO animals (*p* = 0.0060). **(B)** Representative current traces from non-cholinergic neurons of the WT (*Kcnq4^+/+^*, *black*) and KO (*Kcnq4*^–/–^, *purple*) samples elicited by the same voltage protocol as in panel **(A)**. **(C)** Statistical comparison of the relaxation current of cholinergic neurons at –40 mV from the WT (+/+; *n* = 13) and KO (–/–; *n* = 8) samples (*green*: the M-current exists; *red*: no M-current detected; *white squares*: mean ± SD). The average relaxation current at –40 mV was 42.57 ± 7.95 pA in the WT and 14.85 ± 6.12 pA in the KO animals (*p* = 0.0133). *Dotted lines* indicate 0 pA. All non-cholinergic neurons lacked the M-current. **(D,E)** Representative voltage traces obtained from the WT (*Kcnq4^+/+^*, *black*) and KO (*Kcnq4*^–/–^, *purple*) samples from cholinergic **(D)** and non-cholinergic **(E)** neurons elicited by a 1-s-long square current pulse with 100 pA amplitude. *Dotted lines* indicate 0 mV. **(F)** Statistical comparison of the adaptation index (AI) of the cholinergic (*ChAT pos.*) and non-cholinergic (*ChAT neg.*) neurons from the WT (+/+) and KO (–/–) samples (*gray circles*: individual data; *white squares*: mean ± SD). *p < 0.050; **p < 0.010 (*n* = 9 for WT cholinergic, *n* = 6 for KO cholinergic, and *n* = 5 for WT and KO non-cholinergic neurons). For cholinergic cells, the AIs were 0.34 ± 0.02 in the WT and 0.23 ± 0.04 in KO mice (*p* = 0.0399; *n* = 6 for KO and *n* = 9 for WT). For non-cholinergic cells, the AIs were 0.28 ± 0.06 in the WT and 0.11 ± 0.11 in KO mice (*p* = 0.0094; *n* = 5 for both).

As the KCNQ channel subunits exhibited alterations in their expressions, we then studied the electrical properties of PPN neurons from KCNQ4 KO animals by electrophysiology. Firstly, we tested for the presence of the M-current in these neurons. To determine neuron identity, we labeled the recorded neurons with biocytin and checked their cholinergic nature with *post*-*hoc* ChAT immunohistochemistry. The current recorded at –20 mV holding potential was significantly lower in KO than in WT animals, indicating an average reduction of the M-current (Figures 4A–C). In addition, we determined that the M-current was absent (<10 pA at –40 mV) in 62.5% of the KO cases, while WT animals only exhibited its absence in 7.7% of cases (Figure 4C). The reduction in the average current recorded at –20 mV was due to the total absence of the M-current in the population described above. The M-current was not recorded in ChAT-negative neurons, and neither in WT (*n* = 5) nor in KO (*n* = 5) animals (Figure 4B).

As the M-current exerts an important action on the SFA, being capable of determining the synchronization of the neighboring neurons, we analyzed whether the absence of a KCNQ4-mediated M-current can affect the SFA. We elicited trains of action potentials by depolarizing square current injections and then calculated the AIs of the trains. The AIs of the cholinergic neurons from KCNQ4 KO animals were significantly lower than those of the WT (Figures 4D,F). In agreement with the experiments shown above, 66.67% of the KO neurons exhibited lower AIs. We also studied the SFA for the non-cholinergic neurons from KCNQ4 KO mice. As expected, on the basis of our previous results, the AIs for the cholinergic neurons were significantly lower than those of the non-cholinergic ones. Interestingly, a marked reduction of the AIs was seen in KO compared to WT mice (0.28 ± 0.06 in WT and 0.11 ± 0.11 in KO, *p* = 0.0094; Figures 4E,F).

In summary, these experiments showed that KCNQ4 is important in forming functional KCNQ channels in a subset of PPN neurons, which generates the M-current. This alteration impacts on the neuronal firing pattern by changing the SFA in cholinergic and non-cholinergic neurons.

#### Presence of Functional KCNQ4 Subunits in the PPN

**FIGURE 5:**
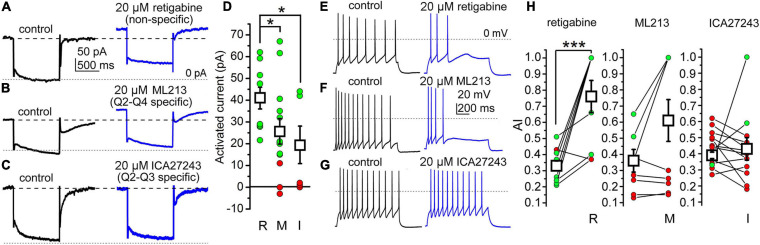
The presence of the KCNQ4 subunit can be detected in a subpopulation of PPN cholinergic neurons. **(A)** Actions of the nonspecific KCNQ opener retigabine on the M-current (*black*: control trace; *blue*: trace with retigabine). **(B)** Representative M-current traces under control conditions (*black*) and with the KCNQ2- and KCNQ4-specific opener ML213 (*blue*). **(C)** Representative M-current traces under control conditions (*black*) and with the KCNQ2- and KCNQ3-specific opener ICA27243 (*blue*). *Dashed lines* indicate the holding currents in the control. *Dotted lines* indicate 0 pA. **(D)** Statistical comparisons of the currents activated by retigabine (*R*), ML213 (*M*), and ICA27243 (*I*) at –20 mV holding potential (*green*: outward currents greater than 10 pA; *red*: currents less than 10 pA; *black squares*: mean ± SD). With retigabine, the holding current at –20 mV increased from 95.25 ± 11.96 to 140.23 ± 12.24 pA (*n* = 11, *p* = 0.0089). With ML213, the increase of the holding current at –20 mV holding potential was 25.61 ± 5.94 pA (from 143.99 ± 28.00 to 169.61 ± 29.66 pA; *n* = 13, *p* = 0.269). With ICA27243, the increase in the outward current was 19.00 ± 8.61 pA (from 165.04 ± 23.23 to 174.96 ± 25.78 pA; *n* = 7, *p* = 0.389). **(E–G)** Representative voltage traces obtained with 100 pA depolarizing current injections under the control conditions (*black*) and with non-selective (retigabine) *p < 0.050. **(E)**, KCNQ2- and KCNQ4-selective (ML213) **(F)**, and KCNQ2- and KCNQ3-selective (ICA27243) **(G)** openers. *Dotted lines* indicate 0 mV. **(H)** Statistical comparison of the changes in AI by openers with different selectivities (*green*: increase; *red*: no change or decrease in individual data; *black squares*: mean ± SD). ***p < 0.001 (significant difference). With retigabine, the AI increased from 0.330 ± 0.035 to 0.755 ± 0.100 (*n* = 9, *p* = 0.0005), with ML213, the AI changed from 0.357 ± 0.066 to 0.608 ± 0.131 (*n* = 10, *p* = 0.067), and with ICA27243, the AI was 0.392 ± 0.025 in the control and 0.439 ± 0.098 after drug application (*n* = 10, n.s.).

As KCNQ4 participates in KCNQ channel structure in some PPN neurons, we now dissected its contribution to the electrical properties in WT animals by using different subunit-specific KCNQ channel openers. For the control, we performed an analysis of the current recorded at –20 mV holding potential (“holding current”) using 20 μM retigabine, a nonspecific KCNQ channel opener. Retigabine elicited an outward shift of the holding current at –20 mV in 100% of the neurons (Figures 5A,D).

Next, we tested the KCNQ2- and KCNQ4-specific M-current opener ML213 (20 μM). This generated a shift in the holding current in 62.9% of the cholinergic neurons tested; the rest were insensitive to it (Figures 5B,D).

Then, we administered the KCNQ2- and KCNQ3-specific opener ICA27243 (20 μM). In about 57.1% of the cases, this opener did not change the holding current (Figures 5C,D). Signs of activation of the KCNQ channels were seen in 42.9% of the neurons (Table 2).

**TABLE 2:** Actions of M-current openers on the KCNQ subunits.

	Q2	Q3	Q2/Q3	Q4	Q3/Q4	Q5	Q3/Q5	Q4/Q5	Outward current occurred (%)	No outward current (%)	AI increased (%)	No AI change (%)
ML213	X		X	X				X	69.2	30.8	44.4	55.6
ICA27243	X	X	X		X		X		42.8	57.2	20	80

*X denotes KCNQ channel subunits potentially affected by the opener in question. AI, adaptation index.*

In the next series of experiments, we evaluated the effect of the openers on the SFA. With retigabine, we observed an increase of the AI in the vast majority (88.9%) of the cases (Figures 5E,H), indicating that the M-current participates in the SFA of cholinergic neurons, although other channels also contribute. ML213 affected excitability in a smaller number of neurons, as it increased the AI in only 44.4% of the cases (*n* = 10; Figures 5F,H). With ICA27243, the effect on the AI was even smaller: it increased the AI in only 20.0% of all cases (*n* = 10; Figures 5G,H and Table 2).

Taken together, we can state that not all PPN cholinergic neurons possess a KCNQ4-mediated M-current. Based on the percentages of the activated neurons elicited with all openers, and their selectivity, we can assume that only a subpopulation of PPN cholinergic neurons possesses functional KCNQ4 subunits (Table 2).

### Insights into the Role of the M-Current in the PPN

We found that the composition of the KCNQ channels in PPN cholinergic neurons is heterogeneous and that the M-current affects the electrical properties of these neurons. Therefore, lastly, we analyzed the impact of the modulation of the M-current on some PPN functions. As deletion of the KCNQ4 subunit altered the expressions of the other subunits (see above), and as we seek to analyze the function of the M-current as physiologically as possible, we discarded the use of KCNQ4 KO mice for these studies. In these experiments, we used transgenic mice for the identification and/or activation of neuronal populations.

#### M-Current Modulation in Cholinergic Neurons

**FIGURE 6:**
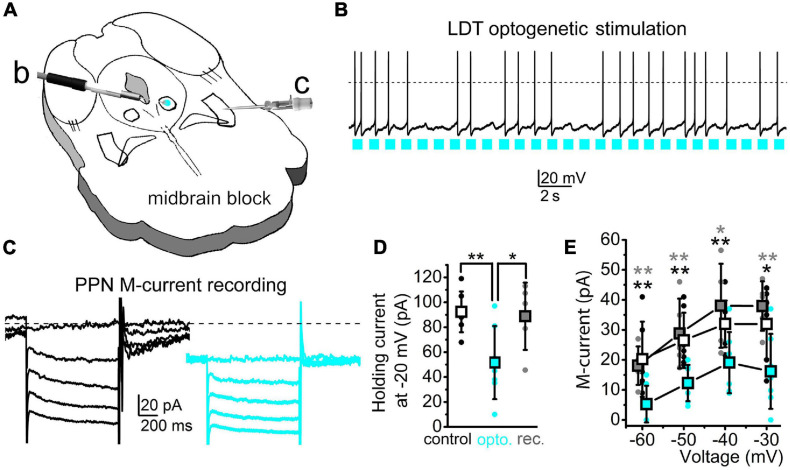
Stimulation of a cholinergic PPN input can effectively reduce the PPN M-current amplitude. **(A)** Schematic drawing of the experimental arrangement. The laterodorsal tegmental nucleus (LDT) of the midbrain block prepared from a ChAT-ChR2 mouse was optogenetically stimulated [*blue spot*: *b*; see panel **(B)**] in parallel with the patch-clamp recording from the PPN [*c*; see panel **(C)**]. **(B)** Optogenetic stimulation of a cholinergic neuron in the LDT with 1 Hz pulsatile blue light (*blue squares*) and the consequential action potential firing of the activated neuron. **(C)** The M-current recorded from a PPN cholinergic neuron before (*black*) and during (*blue*) optogenetic stimulation of the LDT. Neurons were held at –20 mV and 1-s-long repolarizing steps were applied from –30 to –60 mV with a 10-mV decrement. **(D)** Statistical comparison of the holding currents at –20 mV voltage recorded before (*hollow squares* and *black dots*), during optogenetic stimulation (*blue squares* and *blue dots*), and after recovery (*gray squares* and *gray dots*). **p < 0.010. **(E)** Statistical comparison of the repolarizing current steps recorded before (*hollow*), during optogenetic stimulation (*blue*), and after recovery (*gray*). *Squares* represent the mean ± SD and *dots* represent individual data. *p < 0.050; **p < 0.010 (*black asterisks*: significance between control and illuminated; *gray asterisks*: significance between the illuminated and recovered datasets). The decreases by optogenetic stimulation were 44.9 ± 14.9% at –60 mV, 48.5 ± 10.9% at –50 mV, 62.6 ± 12.5% at –40 mV, and 72.7 ± 24.2% at –30 mV (*n* = 7). During recovery, compared to the control, current values were 104.6 ± 22.9% at –60 mV, 101.5 ± 4.8% at –50 mV, 113.3 ± 12.3% at –40 mV, and 128.3 ± 24% at –30 mV (*n* = 5). Differences between the control and illuminated and between the illuminated and recovered datasets were statistically significant in all cases (*p* = 0.0026–0.0253).

We aimed to test whether a near-physiological activation of a cholinergic input of the PPN neurons like the LDT can effectively inhibit the M-current in PPN cholinergic neurons. In this experiment, the LDT of ChAT-channelrhodopsin2 (ChR2) mice was optogenetically stimulated with 1 Hz frequency in a 500-μm-thick coronal brainstem block while the M-current of PPN cholinergic neurons was recorded (Figures 6A–C). Light pulses on LDT cholinergic neurons elicited action potential in almost 80.0% of the cases (Figure 6B). The stimulation of LDT activity induced a decrease in the holding current of PPN neurons to 60.5 ± 6.5% of the control (Figures 6C,D). Five minutes after finishing illumination, the holding current is back to similar values prior to the illumination (*p* = 0.0037 and 0.0259, respectively; Figure 6D). The M-current also significantly decreased at all voltages tested. After illumination, the M-current decreased in a voltage-dependent manner (Figure 6E). After 5-min recovery, the M-current returned to control values at all voltages tested (Figure 6E). We concluded that optogenetic activation of a single cholinergic input of the PPN can effectively inhibit the M-current, indicating that the modulation of this current could regulate PPN function.

#### Effects of the M-Current on Cholinergic Neuronal Synchronization

**FIGURE 7:**
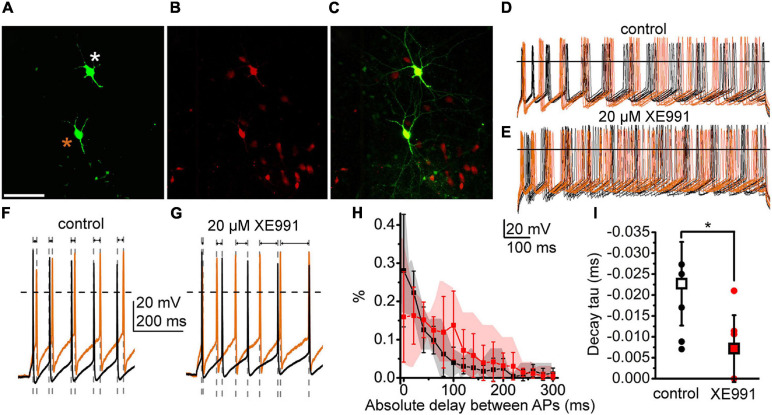
The M-current of PPN cholinergic neurons can control the synchronization of the neighboring neurons. **(A–C)** Two cholinergic neurons labeled in the PPN during recordings (*asterisks*). **(A)** Biocytin labeling (*scale bar*, 100 μm). **(B)** ChAT-dependent tdTomato expression. **(C)** Merged image. **(D,E)** Action potential series elicited by 100 pA depolarizing stimuli from the two neighboring neurons under the control conditions **(D)** (*brown traces*: recordings from the neuron labeled with *brown asterisk*; *black traces*: recorded from the neuron labeled with *white asterisk*) and in the presence of the M-current inhibitor XE991 **(E)**. **(F,G)** Representative pairs of traces taken from panels **(D,E)** at high time resolution under the control conditions **(F)** and with XE991 **(G)** representing changes of the delay intervals between spikes (indicated with *gray dashed lines* and *arrows*). **(H)** Mean histograms of absolute delays between the action potentials recorded from the neighboring neurons with 20-ms bins (mean ± SD; *black*: control; *red*: with XE991; *gray* and *pink clouds*: ranges of individual data). **(I)** Decay tau values obtained with the fit of the individual histograms of absolute delays under the control conditions (*hollow square* and *black dots*) and with XE991 (*red square red dots*). *Squares* represent means and *dots* represent individual data.

As synchronization in firing is a functional property of neighboring PPN neurons, finally, we analyzed whether the M-current participates in this process. To modulate the M-current, we used the KCNQ channel blocker XE991 and recorded the response of two synaptically non-coupled neurons depolarized simultaneously with 100 pA (Figures 7A–C). Administration of XE991 to the tissue decreased the AI of the recorded neurons from 0.353 ± 0.026 to 0.277 ± 0.028 (*p* = 0.0375; not shown), the same as in KCNQ4 KO PPN cholinergic neurons, indicating the action of the drug. We recorded action potential trains and assessed time differences between the closest action potentials (Figures 7D,E). For the neuron pair in the control conditions, we observed a high number of short delay values, while in the presence of XE991, the longer delay values with greater standard deviations were seen (Figures 7F–H). In the control, more than 50.0% of the delay values were shorter than 40 ms. When XE991 is present, the delay values were more uniformly distributed for the first 100 ms (Figure 7H). The decay tau of the distribution histograms significantly decreased from –0.022 ± 0.010 to –0.007 ± 0.008 (*p* = 0.0302; Figure 7I). In consequence, action potential firing of the neighboring neurons in the presence of the M-current blocker was less synchronized.

With these results, we confirmed our previous findings that the M-current exists in the cholinergic but is absent in the non-cholinergic neurons of the PPN. We also showed that a mostly somatic M-current can be effectively inhibited by the physiological cholinergic inputs of the nucleus. Blockade of the M-current causes loss of synchronization between neighboring neurons.

## Discussion

In the present project, we showed that the deletion of the KCNQ4 subunit leads to mild disturbances in the adaptation of the circadian rhythm to changes of environmental light. These changes are partially modulated by the cholinergic neurons of the pedunculopontine nucleus. In a subpopulation of them, we showed that functional KCNQ4 subunits are present. Deletion of this subunit might affect the presence and function of the other KCNQ subunits and the potassium channels. We also showed that regulation of PPN activity *via* the M-current takes place with the activation of nuclei providing cholinergic inputs for the PPN. Under experimental circumstances, M-current inhibition desynchronizes neighboring cholinergic neurons.

We found that deletion of the KCNQ4 subunit, partially due to its expression in the PPN, caused disturbances in the adaptation to alterations in the circadian rhythm. In complete darkness, KCNQ4 KO mice increased the activity time, accompanied with longer distances ran under this condition, with a greater standard deviation of the dataset compared to their WT littermates. In consequence, KCNQ4 KO mice showed a lower capacity to regulate the sleep–wakefulness cycle upon switching from alternating light–darkness cycles to full darkness, suggesting a contribution to the regulation of RAS functions. In 2-month-old animals, hearing loss was already present, but was less prominent than that in older ones (Carignano et al., 2019); thus, the actions seen on activity cycles are potentially at least partially due to the lack of the ion channel subunit in brainstem structures. In addition, there was no significant difference in the activity cycles and movement in alternating LD conditions. Changes in outer light conditions possibly do not affect hearing and tactile sensation, rather the structures regulating the activity cycles. It is also known that the raphe nuclei and the VTA have functional KCNQ4 subunits that affect the neuronal functions in these regions (Zhao et al., 2017; McGuier et al., 2018; Su et al., 2019). One can hypothesize that these structures, as they are members of the RAS, contribute to the alterations in activity cycles found in KCNQ4 KO animals. The PPN, raphe nuclei, and VTA are all involved in both sleep–wakefulness and movement regulation (Monti, 2010; Mena-Segovia and Bolam, 2017; Zhao et al., 2017; MacLaren et al., 2018; McGuier et al., 2018; Jing et al., 2019; Su et al., 2019). Therefore, as was expected, the distances ran under different environmental light conditions were significantly different in WT and KO animals. Based on our findings and literature data (Kroeger et al., 2017), we can conclude that potassium channels composed of KCNQ4 subunits in the RAS including the PPN are modulating activity cycles and movement.

The main pathology generated by KCNQ4 malfunction is the progressive hearing loss, DFNA2. Although it is considered as non-syndromic, Heidenreich et al. (2011) demonstrated another symptom. They showed an enhancement of tactile sensation in both animal models and DFNA2 patients. Our results suggest that, besides the dominating hearing loss and the additional changes of tactile sensation, there are further potential symptoms of DFNA2. Symptoms related to alterations in the activity cycles and movement could also be present in DFNA2 patients, but to the best of our knowledge, these have not been described yet. Besides, KCNQ4 expression in human CNS has not been investigated so far.

The presence of the M-current was described in the nuclei of the RAS, such as in the VTA and in the raphe nuclei (Drion et al., 2010; Zhao et al., 2017; Su et al., 2019). We have already demonstrated that PPN cholinergic neurons possess the M-current, and this contributes to modulating the SFA and the other excitability-related parameters (Bordas et al., 2015). Based on experiments using genetically identified cholinergic and GABAergic neurons, we demonstrated that the presence of the M-current is a functional marker of cholinergic neurons (Bordas et al., 2015). Currently, we have added data from genetically identified glutamatergic neurons and immunohistochemical labeling of the KCNQ subunits and confirmed our previous statement. We found that different KCNQ subunits are present in the PPN at the mRNA and protein levels. With the exception of KCNQ2, the other KCNQ subunits co-localized with ChAT labeling. This finding supports the functional results that the M-current is present in cholinergic neurons but absent in non-cholinergic ones. The KCNQ channels in the PPN would be composed of either KCNQ3 and KCNQ4, KCNQ3 and KCNQ5, or KCNQ4 and KCNQ5.

Ion channels responsible for neuronal M-current are composed of homomeric KCNQ2 or the combination of KCNQ2 and KCNQ3 subunits (Brown and Passmore, 2009; Soldovieri et al., 2011), and of KCNQ5 in most of the cases (Shah et al., 2002; Huang and Trussell, 2011). Heteromers formed by KCNQ2/KCNQ3 subunits are located in the axon initial segments and nodes of Ranvier, regulating firing (Schwarz et al., 2006; Klinger et al., 2011). KCNQ5 is present in the postsynaptic membranes of hippocampal neurons contributing to network synchronization (Fidzinski et al., 2015) and in presynaptic locations controlling neurotransmitter release (Huang and Trussell, 2011). KCNQ3/KCNQ5 heteromers also exist in the CNS (Jentsch, 2000; Delmas and Brown, 2005). Although the most relevant KCNQ4 locations are in the sensory and peripheral systems (cochlear outer hair cells, DRG, and Merkel’s and Pacinian corpuscules) (Kubisch et al., 1999; Beisel et al., 2005; Heidenreich et al., 2011), it is also in the CNS, with a more restricted pattern compared to the other subunits. Its expression is restricted to the brainstem auditory nuclei and the RAS (Kharkovets et al., 2000). From the latter group of nuclei, the location of the subunit in the VTA and raphe nuclei has been demonstrated earlier with morphological methods, but its functional presence was only recently confirmed (Zhao et al., 2017; McGuier et al., 2018; Su et al., 2019). We used morphological and pharmacological methods, as well as transgenic animals, to demonstrate the presence of the KCNQ4 subunit in the PPN. All methods uniformly showed that not all PPN cholinergic neurons, but a subset of them, expressed KCNQ4. This finding is similar to that shown by Heidenreich et al. (2011) in the DRG, where also only a fraction of the neurons was positive to KCNQ4, and in the dorsal raphe (Zhao et al., 2017).

We observed that KCNQ4-possessing cholinergic neurons are rather located in the caudal PPN. This observation strengthens the view that PPN neurons are far from uniform, but have several functional subgroups in terms of membrane properties (Baksa et al., 2019). Furthermore, the rostral PPN (known as pars dissipata) differs from the caudal part (pars compacta) in efferent connectivity, as rostrally located cholinergic neurons project to the dorsolateral striatum and substantia nigra pars compacta, whereas the caudally located ones project to the VTA and the dorsomedial striatum (Martinez-Gonzalez et al., 2011). Different parts of the PPN might serve different functions, as the rostral PPN is rather involved in motor functions (Alderson et al., 2008; Mena-Segovia, 2016) whereas the caudal PPN plays a role in learning and attention (Inglis et al., 2001; Wilson et al., 2009). This might imply that KCNQ4-possessing cholinergic neurons can contribute to the latter functions. However, further studies are needed to determine the contribution of the PPN to the observed changes and to what extent it affects the obvious changes related to motor functions. It has also not been described whether KCNQ4-expressing cholinergic neurons have roles different from those of KCNQ4-negative ones.

Moreover, we determined the expression of KCNQ3 and KCNQ5 in PPN cholinergic neurons, but were not able to determine this in the same neurons as KCNQ4. However, the fact that KCNQ4 KO mice changed KCNQ3 expression and a high proportion of these neurons lacked the M-current opens the possibility to form functional heteromers with KCNQ3 and/or KCNQ5. In this regard, we speculate that the presence of the KCNQ4 subunit is characteristic of certain sensory pathways and nuclei modulating them for adaptation to the sleep–wakefulness cycle (Kharkovets et al., 2000; Heidenreich et al., 2011).

A noteworthy discrepancy was seen between the proportion of neurons possessing KCNQ4 subunits (9.0%) and the number of neurons having functional KCNQ4 subunits revealed by subunit-specific openers (14.0–27.0%), as well as the proportion of neurons lacking the M-current in KCNQ4 KO mice (62.5%). The percentages of neurons with functional KCNQ4 subunits were calculated from the differences in the proportions of neurons responding to KCNQ2/KCNQ4- and KCNQ2/KCNQ3-specific openers by the occurrence of outward currents (27%) or by an increase in AI (14%) (Table 2). In WT animals, the differences in positivity between 9 and 14–27% could be due to the various technique resolutions and limitations of the methods. A possible limitation of the pharmacological study is that the applied openers might exert some weaker actions on the other KCNQ subunits than on the ones they are thought to be selective for. We cannot fully exclude that ML213 (KCNQ2- and KCNQ4-specific opener) caused some activation in the KCNQ5 subunit as well, therefore leading to a moderate overestimation of the proportion of KCNQ4-possessing cholinergic neurons (Brueggemann et al., 2014). The greater differences between the WT and KO genotypes (9–27 and 62.5%, respectively) are probably due to the altered expressions of the other KCNQ subunits and probably other potassium channels in KCNQ4 KO mice.

Another possible reason for the differences in the percentages of KCNQ4-positive neurons found by immunohistochemistry and electrophysiology is the age range of the experimental animals. For electrophysiology, juvenile mice with almost no progress in hair cell loss were employed, whereas adult ones with great progress of hearing loss were used for the morphological studies. In addition, behavioral tests were performed on young adult mice with some progress in hearing loss. Using mice of different ages due to technical reasons raises the question of whether these results show different time points of the progress in a neurodegenerative process.

We did not find age dependence of the recorded parameters within the age ranges in the experiments. Furthermore, in the outer and inner hair cells and the spiral ganglion neurons of 1-year-old animals, no further neuronal loss has been reported before in KCNQ4 KO mice (Heidenreich et al., 2011; Zhao et al., 2017; Carignano et al., 2019). Although a reduction in neuronal numbers in the brainstem has not been deeply analyzed, we found no significant reduction in PPN cholinergic neurons in the 6-month-old KO animals compared to the WT.

In conclusion, loss of KCNQ4 does not seem to cause neurodegeneration of the PPN, neither by excitotoxicity due to the lack of a hyperpolarizing current nor *via* the consequence of a hearing deficit. However, the different ages of mice used for the different experiments might serve as a limitation in the interpretation of our findings.

In KCNQ4 KO mice, as expected, the KCNQ4 subunit disappeared both at the mRNA and protein levels. In parallel with these changes, the levels of KCNQ2 and KCNQ5 remained unchanged, but KCNQ3 was upregulated. Interestingly, the KCNQ3 subunit changed its expression pattern from cholinergic to non-cholinergic neurons. We did not find the M-current in the last ones, indicating that KCNQ3 does not form functional channels or that they may form homomeric channels with very low membrane expression in non-cholinergic neurons (Brown and Passmore, 2009). However, we determined alterations in the electrical properties of these neurons.

Besides the alterations found in the expressions of the KCNQ subunits in KO animals, probably other potassium channels were affected as well. The non-cholinergic neurons of WT mice had significantly smaller AIs than the cholinergic ones. As expected, deletion of the KCNQ4 subunit significantly reduced the AIs of cholinergic neurons. However, quite unexpectedly, the AIs were also significantly reduced in the non-cholinergic neurons of the KO animals compared to the WT. As other potassium (and chloride) channels out of the M-current also determine the AI (Ha and Cheong, 2017), we suppose that the deletion of KCNQ4 affected the expression and function of several voltage-gated potassium channels.

Alterations in the expression patterns of the KCNQ channel subunits were observed in different tissues during pathologies such as hypertension, vascular tumors, or retinal degeneration, which in turn contribute to alterations in cellular properties (Jepps et al., 2011; Caminos et al., 2015; Serrano-Novillo et al., 2020). We determined a similar behavior when disturbing KCNQ4 expression, which altered the gene expression and protein localization of the other KCNQ channel subunits and the proportion of neurons lacking the M-current. In conclusion, these observations raise the possibility that KCNQ4 is important not only as one of the subunits forming channels for the M-current but also might be a potential regulator of the expression of other M-current subunits, setting their physiological function. In agreement with this, RAS-related behavioral alterations were detected in KCNQ4 KO mice (see above).

We have previously shown that the SFA is critically affected by the M-current (Bordas et al., 2015). Here, in accordance with modeling and data from other brain areas (Leão et al., 2009; Roach et al., 2015, 2016), we showed that the SFA helps in the synchronization of neuronal populations. Blockade of the M-current decreased the SFA, which decreased synchronization between two neighboring neurons. We also showed that the M-current is effectively inhibited by the cholinergic inputs of the PPN. However, an important caveat is that the actions on the SFA were recorded on *ex vivo* preparations at room temperature. Changes in synchronization by the inhibition or activation of the M-current might be different in an intact brain at physiological body temperatures. Furthermore, stimulation of a cholinergic input does not only mean release of acetylcholine in the target structures but also of nitric oxide (Veleanu et al., 2016), which might also contribute to the inhibition of the M-current (Ooi et al., 2013).

Taken together, it seems likely that cholinergic activation of a cholinergic nucleus desynchronizes its neuronal population. As the PPN provides local axon collaterals for itself, innervates the contralateral PPN, and receives cholinergic fibers from the LDT, cholinergic activation might spread to all mesopontine cholinergic structures and contribute to the desynchronization of cholinergic neuronal populations (Honda and Semba, 1995; Mena-Segovia et al., 2008). Desynchronization of PPN units takes place in parallel with cortical desynchronization (Mena-Segovia et al., 2008; Boucetta et al., 2014; Petzold et al., 2015). One can assume that cholinergic inhibition of the M-current contributes to the autoregulatory desynchronization of cholinergic structures and, in turn, to the regulation of global brain states.

We conclude that the KCNQ4-dependent M-current of the RAS might have a modulatory role in the adaptation of the activity cycles to environmental changes and in the related changes in motor activity. The PPN contributes to regulating the sleep–wakefulness cycle and the motor activity, and its M-current, as a modulatory current, may have an important role in these functions. In fact, different roles were determined for the three main neuronal populations of the PPN in the transitions between brain states (Kroeger et al., 2017). Although KCNQ4 is not the only KCNQ subunit present in the nucleus, it has a significant contribution to the function of the M-current. As KCNQ4 is selectively expressed in certain brainstem nuclei, it could be a pharmacological target for the selective modulation of the sleep–wakefulness cycle. KCNQ4-specific openers were hypothesized as effective drugs for the treatment of psychiatric diseases (Sotty et al., 2009; Zhao et al., 2017; McGuier et al., 2018; Su et al., 2019). In addition, based on their actions in the PPN, these openers might affect the sleep–wakefulness cycle, as well as having the potential to slow the progression of neurodegenerative diseases affecting the cholinergic neurons of the PPN, such as progressive supranuclear palsy (MacLaren et al., 2018; Sébille et al., 2019) and Parkinson’s disease (Chambers et al., 2020). They might also help in the adaptation to artificial alterations of the circadian rhythm (e.g., jet lag or space travel) (Brainard et al., 2016). Furthermore, one can also hypothesize that DFNA2 is possibly not a non-syndromic hearing loss, as alterations in the sleep–wakefulness cycle and movement regulation might be seen. These latter hypotheses need further confirmation by clinical studies.

## Data Availability Statement

The original contributions presented in the study are included in the article/supplementary material, further inquiries can be directed to the corresponding author/s.

## Ethics Statement

The animal study was reviewed and approved by Committee of Animal Research of the University of Debrecen (6/2011/DEMÁB; 5/2015/DEMÁB; and 19/2019/DEMÁB) and Universidad Nacional del Sur (083/2016).

## Author Contributions

TB, AK, AC, and KP performed patch clamp and optogenetic experiments and contributed to genotyping and maintenance of transgenic strains. SS and LD performed qPCR and immunohistochemical (IHC) experiments. PS and BP performed the activity wheel test. TB, SS, LD, GS, and BP wrote the manuscript. BP supervised the patch clamp and behavioral studies. GS supervised the qPCR and IHC studies. All authors contributed to the article and approved the submitted version.

## Conflict of Interest

The authors declare that the research was conducted in the absence of any commercial or financial relationships that could be construed as a potential conflict of interest.

## Publisher’s Note

All claims expressed in this article are solely those of the authors and do not necessarily represent those of their affiliated organizations, or those of the publisher, the editors and the reviewers. Any product that may be evaluated in this article, or claim that may be made by its manufacturer, is not guaranteed or endorsed by the publisher.

## Footnotes

1
www.circadian.org

## Abbreviations

PPN: pedunculopontine nucleus
RAS: reticular activating system
CNS: central nervous system
LD: light–darkness
VTA: ventral tegmental area
DRG: dorsal root ganglia
LDT: laterodorsal tegmental nucleus
SWS: slow-wave sleep
PS: paradoxical sleep
W: wakefulness
KO: knockout
DD: constant darkness
qPCR: quantitative PCR
cDNA: complementary DNA
SFA: spike frequency adaptation
ChAT: choline acetyltransferase
ChR2: channelrhodopsin-2.

## References

[B1] Alderson H. L. Latimer M. P. Winn P. (2008). A functional dissociation of the anterior and posterior pedunculopontine tegmental nucleus: excitotoxic lesions have differential effects on locomotion and the response to nicotine. *Brain Struct. Funct.* 213 247–253. 10.1007/s00429-008-0174-4 18266007 PMC2522332

[B2] Baksa B. Kovács A. Bayasgalan T. Szentesi P. Köszeghy Á. Szücs P. (2019). Characterization of functional subgroups among genetically identified cholinergic neurons in the pedunculopontine nucleus. *Cell Mol. Life Sci.* 76 2799–2815. 10.1007/s00018-019-03025-4 30734834 PMC6588655

[B3] Beisel K. W. Rocha-Sanchez S. M. Morris K. A. Nie L. Feng F. Kachar B. (2005). Differential expression of KCNQ4 in inner hair cells and sensory neurons is the basis of progressive high-frequency hearing loss. *J. Neurosci.* 25 9285–9293. 10.1523/JNEUROSCI.2110-05.2005 16207888 PMC6725753

[B4] Bordas C. Kovacs A. Pal B. (2015). The M-current contributes to high threshold membrane potential oscillations in a cell type-specific way in the pedunculopontine nucleus of mice. *Front. Cell. Neurosci.* 9:121. 10.3389/fncel.2015.00121 25904846 PMC4388076

[B5] Boucetta S. Cissé Y. Mainville L. Morales M. Jones B. E. (2014). Discharge profiles across the sleep-waking cycle of identified cholinergic, GABAergic, and glutamatergic neurons in the pontomesencephalic tegmentum of the rat. *J. Neurosci.* 34 4708–4727. 10.1523/JNEUROSCI.2617-13.2014 24672016 PMC3965793

[B6] Brainard G. C. Barger L. K. Soler R. R. Hanifin J. P. (2016). The development of lighting countermeasures for sleep disruption and circadian misalignment during spaceflight. *Curr. Opin. Pulm. Med.* 22 535–544. 10.1097/MCP.0000000000000329 27607152

[B7] Brown D. A. Adams P. R. (1980). Muscarinic suppression of a novel voltage-sensitive K+ current in a vertebrate neurone. *Nature* 283 673–676. 10.1038/283673a0 6965523

[B8] Brown D. A. Passmore G. M. (2009). Neural KCNQ (Kv7) channels. *Br. J. Pharmacol.* 156 1185–1195. 10.1111/j.1476-5381.2009.00111.x 19298256 PMC2697739

[B9] Brueggemann L. I. Haick J. M. Cribbs L. L. Byron K. L. (2014). Differential activation of vascular smooth muscle Kv7.4, Kv7.5, and Kv7.4/7.5 channels by ML213 and ICA-069673. *Mol. Pharmacol.* 86 330–341. 10.1124/mol.114.093799 24944189 PMC4152906

[B10] Caminos E. Vaquero C. F. Martinez-Galan J. R. (2015). Relationship between rat retinal degeneration and potassium channel KCNQ5 expression. *Exp. Eye Res.* 131 1–11. 10.1016/j.exer.2014.12.009 25499209

[B11] Carignano C. Barila E. P. Rías E. I. Dionisio L. Aztiria E. Spitzmaul G. (2019). Inner hair cell and neuron degeneration contribute to hearing loss in a DFNA2-like mouse model. *Neuroscience* 410 202–216. 10.1016/j.neuroscience.2019.05.012 31102762

[B12] Chambers N. E. Lanza K. Bishop C. (2020). Pedunculopontine nucleus degeneration contributes to both motor and non-motor symptoms of Parkinson’s disease. *Front. Pharmacol.* 10:1494. 10.3389/fphar.2019.01494 32009944 PMC6974690

[B13] De Leenheer E. M. Ensink R. J. Kunst H. P. Marres H. A. Talebizadeh Z. Declau F. (2002). DFNA2/KCNQ4 and its manifestations. *Adv. Otorhinolaryngol.* 61 41–46. 10.1159/000066802 12408061

[B14] Delmas P. Brown D. A. (2005). Pathways modulating neural KCNQ/M (Kv7) potassium channels. *Nat. Rev. Neurosci.* 6 850–862. 10.1038/nrn1785 16261179

[B15] Devaux J. J. Kleopa K. A. Cooper E. C. Scherer S. S. (2004). KCNQ2 is a nodal K^+^ channel. *J. Neurosci.* 24 1236–1244. 10.1523/JNEUROSCI.4512-03.2004 14762142 PMC6793582

[B16] Drion G. Bonjean M. Waroux O. Scuvée-Moreau J. Liégeois J. F. Sejnowski T. J. (2010). M-type channels selectively control bursting in rat dopaminergic neurons. *Eur. J. Neurosci.* 31 827–835. 10.1111/j.1460-9568.2010.07107.x 20180842 PMC2861736

[B17] Fidzinski P. Korotkova T. Heidenreich M. Maier N. Schuetze S. Kobler O. (2015). KCNQ5 K^+^ channels control hippocampal synaptic inhibition and fast network oscillations. *Nat. Commun.* 6:6254. 10.1038/ncomms7254 25649132

[B18] Fodor J. Al-Gaadi D. Czirják T. Oláh T. Dienes B. Csernoch L. (2020). Improved calcium homeostasis and force by selenium treatment and training in aged mouse skeletal muscle. *Sci. Rep.* 10:1707. 10.1038/s41598-020-58500-x 32015413 PMC6997352

[B19] Franklin K. B. J. Paxinos G. (2013). *The Mouse Brain in Stereotaxic Coordinates*, 4th Edn. San Diego, CA: Elsevier.

[B20] Gorman M. R. Elliott J. A. (2003). Entrainment of 2 subjective nights by daily light:dark:light:dark cycles in 3 rodent species. *J. Biol. Rhythms* 18 502–512. 10.1177/0748730403260219 14667151

[B21] Gunthorpe M. J. Large C. H. Sankar R. (2012). The mechanism of action of retigabine (ezogabine), a first-in-class K^+^ channel opener for the treatment of epilepsy. *Epilepsia* 53 412–424. 10.1111/j.1528-1167.2011.03365.x 22220513

[B22] Ha G. H. Cheong E. (2017). Spike frequency adaptation in neurons of the central nervous system. *Exp. Neurobiol.* 26 179–185. 10.5607/en.2017.26.4.179 28912640 PMC5597548

[B23] Hansen H. H. Waroux O. Seutin V. Jentsch T. J. Aznar S. Mikkelsen J. D. (2008). Kv7 channels: interaction with dopaminergic and serotonergic neurotransmission in the CNS. *J. Physiol.* 586 1823–1832. 10.1113/jphysiol.2007.149450 18174210 PMC2375731

[B24] Harrison E. M. Walbeek T. J. Sun J. Johnson J. Poonawala Q. Gorman M. R. (2016). Extraordinary behavioral entrainment following circadian rhythm bifurcation in mice. *Sci. Rep.* 6:38479. 10.1038/srep38479 27929128 PMC5144065

[B25] Heidenreich M. Lechner S. G. Vardanyan V. Wetzel C. Cremens C. W. De Leenheer E. M. (2011). KCNQ4 K^+^ channels tune mechanoreceptors for normal touch sensation in mouse and man. *Nat. Neurosci.* 15 138–145. 10.1038/nn.2985 22101641

[B26] Hernandez C. C. Zaika O. Tolstykh G. P. Shapiro M. S. (2008). Regulation of neural KCNQ channels: signalling pathways, structural motifs and functional implications. *J. Physiol.* 586 1811–1821. 10.1113/jphysiol.2007.148304 18238808 PMC2375728

[B27] Honda T. Semba K. (1995). An ultrastructural study of cholinergic and non-cholinergic neurons in the laterodorsal and pedunculopontine tegmental nuclei in the rat. *Neuroscience* 68 837–853. 10.1016/0306-4522(95)00177-K 8577378

[B28] Huang H. Trussell L. O. (2011). KCNQ5 channels control resting properties and release probability of a synapse. *Nat. Neurosci.* 14 840–847. 10.1038/nn.2830 21666672 PMC3133966

[B29] Inglis W. L. Olmstead M. C. Robbins T. W. (2001). Selective deficits in attentional performance on the 5-choice serial reaction time task following pedunculopontine tegmental nucleus lesions. *Behav. Brain Res.* 123 117–131. 10.1016/s0166-4328(01)00181-4 11399325

[B30] Jentsch T. J. (2000). Neuronal KCNQ potassium channels: physiology and role in disease. *Nat. Rev. Neurosci.* 1 21–30. 10.1038/35036198 11252765

[B31] Jepps T. A. Chadha P. S. Davis A. J. Harhun M. I. Cockerill G. W. Olesen S. P. (2011). Downregulation of Kv7.4 channel activity in primary and secondary hypertension. *Circulation* 124 602–611. 10.1161/CIRCULATIONAHA.111.032136 21747056

[B32] Jing M. Y. Han X. Zhao T. Y. Wang Z. Y. Lu G. Y. Wu N. (2019). Re-examining the role of ventral tegmental area dopaminergic neurons in motor activity and reinforcement by chemogenetic and optogenetic manipulation in mice. *Metab. Brain Dis.* 34 1421–1430. 10.1007/s11011-019-00442-z 31313126

[B33] Kharkovets T. Dedek K. Maier H. Schweizer M. Khimich D. Nouvian R. (2006). Mice with altered KCNQ4 K^+^ channels implicate sensory outer hair cells in human progressive deafness. *EMBO J.* 25 642–652. 10.1038/sj.emboj.7600951 16437162 PMC1383535

[B34] Kharkovets T. Hardelin J. P. Safieddine S. Schweizer M. El-Amraoui A. Petit C. (2000). KCNQ4, a K^+^ channel mutated in a form of dominant deafness, is expressed in the inner ear and the central auditory pathway. *Proc. Natl. Acad. Sci. U.S.A.* 97 4333–4338. 10.1073/pnas.97.8.4333 10760300 PMC18242

[B35] Klinger F. Gould G. Boehm S. Shapiro M. S. (2011). Distribution of M-channel subunits KCNQ2 and KCNQ3 in rat hippocampus. *Neuroimage* 58 761–769. 10.1016/j.neuroimage.2011.07.003 21787867 PMC3166433

[B36] Koyama S. Appel S. B. (2006). Characterization of M-current in ventral tegmental area dopamine neurons. *J. Neurophysiol.* 96 535–543. 10.1152/jn.00574.2005 16394077

[B37] Kroeger D. Ferrari L. L. Petit G. Mahoney C. E. Fuller P. M. Arrigoni E. (2017). Cholinergic, glutamatergic, and GABAergic neurons of the pedunculopontine tegmental nucleus have distinct effects on sleep/wake behavior in mice. *J. Neurosci.* 37 1352–1366. 10.1523/JNEUROSCI.1405-16.2016 28039375 PMC5296799

[B38] Kubisch C. Schroeder B. C. Friedrich T. Lütjohann B. El-Amraoui A. Marlin S. (1999). KCNQ4, a novel potassium channel expressed in sensory outer hair cells, is mutated in dominant deafness. *Cell* 96 437–446. 10.1016/s0092-8674(00)80556-5 10025409

[B39] Leão R. N. Tan H. M. Fisahn A. (2009). Kv7/KCNQ channels control action potential phasing of pyramidal neurons during hippocampal gamma oscillations in vitro. *J. Neurosci.* 29 13353–13364. 10.1523/JNEUROSCI.1463-09.2009 19846723 PMC6665214

[B40] Linley J. E. Pettinger L. Huang D. Gamper N. (2012). M channel enhancers and physiological M channel block. *J. Physiol.* 590 793–807. 10.1113/jphysiol.2011.223404 22155935 PMC3381311

[B41] Livak K. J. Schmittgen T. D. (2001). Analysis of relative gene expression data using real-time quantitative PCR and the 2(-Delta Delta C(T)) Method. *Methods* 25 402–408. 10.1006/meth.2001.1262 11846609

[B42] MacLaren D. A. A. Ljungberg T. L. Griffin M. E. (2018). Pedunculopontine tegmentum cholinergic loss leads to a progressive decline in motor abilities and neuropathological changes resembling progressive supranuclear palsy. *Eur. J. Neurosci.* 48 3477–3497. 10.1111/ejn.14212 30339310

[B43] Marrion N. V. (1997). Control of M-current. *Annu. Rev. Physiol.* 59 483–504. 10.1146/annurev.physiol.59.1.483 9074774

[B44] Martinez-Gonzalez C. Bolam J. P. Mena-Segovia J. (2011). Topographical organization of the pedunculopontine nucleus. *Front. Neuroanat.* 5:22. 10.3389/fnana.2011.00022 21503154 PMC3074429

[B45] McGuier N. S. Rinker J. A. Cannady R. Fulmer D. B. Jones S. R. Hoffman M. (2018). Identification and validation of midbrain Kcnq4 regulation of heavy alcohol consumption in rodents. *Neuropharmacology* 138 10–19. 10.1016/j.neuropharm.2018.05.020 29775679 PMC6054890

[B46] Mena-Segovia J. (2016). Structural and functional considerations of the cholinergic brainstem. *J. Neural Transm. (Vienna)* 123 731–736. 10.1007/s00702-016-1530-9 26945862

[B47] Mena-Segovia J. Bolam J. P. (2017). Rethinking the pedunculopontine nucleus: from cellular organization to function. *Neuron* 94 7–18. 10.1016/j.neuron.2017.02.027 28384477

[B48] Mena-Segovia J. Sims H. M. Magill P. J. Bolam J. P. (2008). Cholinergic brainstem neurons modulate cortical gamma activity during slow oscillations. *J. Physiol.* 586 2947–2960. 10.1113/jphysiol.2008.153874 18440991 PMC2517196

[B49] Monti J. M. (2010). The role of dorsal raphe nucleus serotonergic and non-serotonergic neurons, and of their receptors, in regulating waking and rapid eye movement (REM) sleep. *Sleep Med. Rev.* 14 319–327. 10.1016/j.smrv.2009.10.003 20153670

[B50] Nie L. (2008). KCNQ4 mutations associated with nonsyndromic progressive sensorineural hearing loss. *Curr. Opin. Otolaryngol. Head Neck Surg.* 16 441–444. 10.1097/MOO.0b013e32830f4aa3 18797286 PMC2743278

[B51] Ooi L. Gigout S. Pettinger L. Gamper N. (2013). Triple cysteine module within M-Type K^+^ channels mediates reciprocal channel modulation by nitric oxide and reactive oxygen species. *J. Neurosci.* 33 6041–6046. 10.1523/JNEUROSCI.4275-12.2013 23554485 PMC3664272

[B52] Petzold A. Valencia M. Pál B. Mena-Segovia J. (2015). Decoding brain state transitions in the pedunculopontine nucleus: cooperative phasic and tonic mechanisms. *Front. Neural Circuits* 9:68. 10.3389/fncir.2015.00068 26582977 PMC4628121

[B53] Roach J. P. Ben-Jacob E. Sander L. M. Zochowski M. R. (2015). Formation and dynamics of waves in a cortical model of cholinergic modulation. *PLoS Comput. Biol.* 11:e1004449. 10.1371/journal.pcbi.1004449 26295587 PMC4546669

[B54] Roach J. P. Sander L. M. Zochowski M. R. (2016). Memory recall and spike-frequency adaptation. *Phys. Rev. E* 93:052307. 10.1103/PhysRevE.93.052307 27300910 PMC4911895

[B55] Schmittgen T. D. Livak K. J. (2008). Analyzing real-time PCR data by the comparative C(T) method. *Nat. Protoc.* 3 1101–1108. 10.1038/nprot.2008.73 18546601

[B56] Schwarz J. R. Glassmeier G. Cooper E. C. Kao T. C. Nodera H. Tabuena D. (2006). KCNQ channels mediate IKs, a slow K^+^ current regulating excitability in the rat node of Ranvier. *J. Physiol.* 573(Pt 1) 17–34. 10.1113/jphysiol.2006.106815 16527853 PMC1779690

[B57] Sébille S. B. Rolland A. S. Faillot M. Perez-Garcia F. Colomb-Clerc A. Lau B. (2019). Normal and pathological neuronal distribution of the human mesencephalic locomotor region. *Mov. Disord.* 34 218–227. 10.1002/mds.27578 30485555

[B58] Serrano-Novillo C. Oliveras A. Ferreres J. C. Condom E. Felipe A. (2020). Remodeling of Kv7.1 and Kv7.5 expression in vascular tumors. *Int. J. Mol. Sci.* 21:6019. 10.3390/ijms21176019 32825637 PMC7503939

[B59] Shah M. Mistry M. Marsh S. J. Brown D. A. Delmas P. (2002). Molecular correlates of the M-current in cultured rat hippocampal neurons. *J. Physiol.* 544(Pt 1) 29–37. 10.1113/jphysiol.2002.028571 12356878 PMC2290582

[B60] Soldovieri M. V. Miceli F. Taglialatela M. (2011). Driving with no brakes: molecular pathophysiology of Kv7 potassium channels. *Physiology (Bethesda)* 26 365–376. 10.1152/physiol.00009.2011 22013194

[B61] Sotty F. Damgaard T. Montezinho L. P. Mork A. Olsen C. K. Bundgaard C. (2009). Antipsychotic-like effect of retigabine [N-(2-amino-4-fluorobenzylamino)-phenyl)carbamic acid ester], a KCNQ potassium channel opener, via modulation of mesolimbic dopaminergic neurotransmission. *J. Pharmacol. Exp. Ther.* 328 951–962. 10.1124/jpet.108.146944 19098162

[B62] Spitzmaul G. Tolosa L. Winkelman B. H. J. Heidenreich M. Frens M. A. Chabbert C. (2013). Vestibular role of KCNQ4 and KCNQ5 K^+^ channels revealed by mouse models. *J. Biol. Chem.* 288 9334–9344. 10.1074/jbc.M112.433383 23408425 PMC3611004

[B63] Su M. Li L. Wang J. Sun H. Zhang L. Zhao C. (2019). Kv7.4 channel contribute to projection-specific auto-inhibition of dopamine neurons in the ventral tegmental area. *Front. Cell. Neurosci.* 13:557. 10.3389/fncel.2019.00557 31920557 PMC6930245

[B64] Veleanu M. Axen T. E. Kristensen M. P. Kohlmeier K. A. (2016). Comparison of bNOS and chat immunohistochemistry in the laterodorsal tegmentum (LDT) and the pedunculopontine tegmentum (PPT) of the mouse from brain slices prepared for electrophysiology. *J. Neurosci. Methods* 263 23–35. 10.1016/j.jneumeth.2016.01.020 26820905

[B65] Wickenden A. D. Krajewski J. L. London B. Wagoner P. K. Wilson W. A. Clark S. (2008). N-(6-chloro-pyridin-3-yl)-3,4-difluoro-benzamide (ICA-27243): a novel, selective KCNQ2/Q3 potassium channel activator. *Mol. Pharmacol.* 73 977–986. 10.1124/mol.107.043216 18089837

[B66] Wilson D. I. G. MacLaren D. A. A. Philip Winn P. (2009). Bar pressing for food: differential consequences of lesions to the anterior versus posterior pedunculopontine. *Eur. J. Neurosci.* 30 504–513. 10.1111/j.1460-9568.2009.06836.x 19614747

[B67] Woolf N. J. Butcher L. J. (2011). Cholinergic systems mediate action from movement to higher consciousness. *Behav. Brain Res.* 221 488–498. 10.1016/j.bbr.2009.12.046 20060422

[B68] Zhao C. Su M. Wang Y. Li X. Zhang Y. Du X. (2017). Selective modulation of K^+^ channel Kv7.4 significantly affects the excitability of DRN 5-HT neurons. *Front. Cell. Neurosci.* 11:405. 10.3389/fncel.2017.00405 29311835 PMC5735115

